# Emotional contagion in dyadic online video conferences—empirical evidence based on self-report and facial expression data

**DOI:** 10.3389/fpsyg.2025.1546303

**Published:** 2025-04-03

**Authors:** Anton K. G. Marx, David F. Sachs, Anne C. Frenzel, Martin T. Schweizer

**Affiliations:** Department of Psychology, Ludwig-Maximilians-Universität München (LMU Munich), Munich, Germany

**Keywords:** emotional contagion, facial expression analysis, online video conferences, social interaction, cross-recurrence quantification analysis

## Abstract

**Introduction:**

Emotional contagion is an essential and prevalent emotional process in social interaction and comprises the transmission of emotions between two or more individuals. The vast majoriy of prior research explored the emotional contagion in face-to-face human interaction. The present study explored the degree to which emotional contagion occurs in dyadic online video conferences, using subjective self-report and automatically coded facial expression data.

**Methods:**

In a lab-based experimental approach, 104 participants (in 52 dyads) interacted via synchronized computers. They were prompted to talk to each other about recent personally relevant experiences that made them angry, happy, and sad (3 conditions). We recorded participants’ emotions by means of automated facial expression analysis and retrospective self-report after each condition.

**Results:**

Our preregistered analyses provided evidence for emotional contagion of all three emotions during the video conferences based on the self-report data. Regarding facially expressed emotions, only joy seemed to be transmitted, while the frequency of facially expressed anger and sadness was generally very low, and did not differ across conditions. We further explored temporal co-occurrences of facially expressed joy through cross-recurrence quantification analysis. Those results showed that both interaction partners’ facial expressions of joy, but not of anger and sadness, co-occurred significantly above chance.

**Discussion:**

Overall, we conclude that emotions can be transmitted across interaction partners during online video interactions, but the face does not seem to be the key channel for those contagion processes, particularly not for negative emotions.

## Introduction

Since the COVID-19 pandemic, many aspects of social life around the world have been moved to digitally supported environments, including learning activities (Correia et al., 2020), work meetings (Karl et al., 2021), or mental health services (Ghaneirad et al., 2021). While on a technical level, these drastic and rapid changes have proven to be feasible and useful alternatives in many instances, their impact on people’s emotional experiences and interpersonal processes remains largely unclear. More specifically, surprisingly little is known about whether and how an individual’s emotions are transmitted to another person when interacting with each other via an online video conference system. In previous research, this transmission has been referred to as emotional contagion (e.g., Hatfield et al., 1994)—a process that has been linked repeatedly with beneficial emotional outcomes in different socially interactive contexts, such as teacher-student classroom interaction (e.g., Frenzel et al., 2009). However, despite the strong practical implications of online video conferences, previous research in this context is scarce, and only two studies provided mixed evidence on the existence of emotional contagion during online video conference interaction (Gvirts et al., 2023; Mui et al., 2018). As of yet, no study known to us investigated emotional contagion based on self-report and facial expression data during authentic dyadic interaction in a standardized paradigm and environment. To close this research gap, we developed a novel lab-based experimental setup using two synchronized computers, a structured dyadic interaction paradigm (with three emotion conditions) via an online video conference application, and measures of both subjectively experienced and facially expressed emotions.

### Emotional contagion as a basic interpersonal process

Emotional contagion describes the largely automatic and unconscious transmission of emotional experiences between two or more individuals, resulting in a shared experience of emotional states (Dezecache et al., 2015; Elfenbein, 2014; Hatfield et al., 1994). Such emotional states are defined as episodes of synchronized changes in interrelated organismic subsystems in reaction to individually significant stimuli. They are characterized by a subjectively experienced affective core as well as emotion-specific cognitive processes, physiological changes, motivational action tendencies, and expressive behaviors (Moors et al., 2013; Scherer, 2005, 2009). On a conceptual level, emotional contagion is thought to be related to empathy, but it can be delineated clearly from this multifarious and often rather broadly and ambiguously defined concept (e.g., Batson, 2009; Coplan, 2011; Cuff et al., 2016; Decety and Jackson, 2004; Hall and Schwartz, 2019; Pinotti and Salgaro, 2019; Zahavi and Rochat, 2015). First, emotional contagion represents a solely affective response instead of cognitive reactions or processes (e.g., perspective-taking). Second, it is thought to be largely automatic and unintentional, unlike intentional interpersonal processes (e.g., imagining being in another person’s situation). Third, emotional contagion does not necessarily involve any behaviorally or verbally expressive reaction toward the other individual (e.g., consoling or comforting). And last, while empathy demands at least some level of self-other distinction and awareness of the other person and their situation, emotional contagion does not require to distinguish between one’s own and the other person’s emotions (Marx, 2020).

The transmission of emotional states from one person to another (e.g., from a speaking to a listening or responding person) in the sense of emotional contagion is thought to be largely based on the imitation and, thus, temporal coordination of postures, movements, vocalizations, or facial expressions (Dezecache et al., 2015; Hatfield et al., 1994, 2014; see Elfenbein, 2014, for a review of different possible mechanisms). It has long been conceptualized as a step-by-step process involving the perception and subsequent imitation of another person’s facial expressions that lead to the subjective experience of the emotional state in the receiving person arising from bottom-up afferent feedback processes (see Coles et al., 2019, 2022). This conceptualization is rooted in the seminal work by Dimberg (1982), Dimberg and Lundquist (1990), Lundqvist and Dimberg (1995) and Dimberg et al. (2000, 2002). In these studies, they used electromyography to measure participants’ facial muscle movements in response to facial expressions of emotions. Overall, they found that participants reacted with distinct facial muscle reactions that corresponded to the facial expressions of emotions that were displayed as stimulus material during the experiments. In most of these studies, facial expressions of joy and anger were used as stimuli to contrast positively versus negatively valenced emotions. However, while some theorists use the term facial mimicry for this process (e.g., Hatfield et al., 2014), it is important to note, that there are more conceptual perspectives on the interpersonal phenomenon of responding with facial expressions to match another person’s facial expressions (e.g., Drimalla et al., 2019; Holland et al., 2021). Among others, Hess and Fischer prominently proposed the so-called social regulator view on facial/emotional mimicry which emphasizes the role of top-down appraisal processes in reaction to another individual’s emotional signals as necessary antecedents of subsequent facial expression imitation and the potential function of such imitation to foster affiliation and rapport between the interacting individuals (e.g., Hess and Fischer, 2013, 2022). For the sake of conceptual clarity, we, thus, refrain from using the term facial mimicry in the present study and, instead, chose to describe this presumably automatic and unconscious transmission process as temporal coordination of facial expressions during the interaction between the participants.

In addition to employing self-report measures, previous studies on emotional contagion have used facial expression data as an expressive behavioral component of emotions. For example, in a study by Olszanowski et al. (2020), participants’ facial muscle activity was assessed using electromyography while watching videos displaying happiness, anger, or sadness portrayed by actors and their results showed that the participants’ facial activity could partially explain their self-reported discrete emotions in the sense of emotional contagion. Overall, the degree of emotional contagion has been linked to various social and emotional outcomes in previous research and it has been reported to have positive effects on the interaction partners and their experiences. For example, in work-related group interaction, it may foster cooperativeness and improve task performance in teams (Barsade, 2002), in romantic relationships and couple interaction, it seems to contribute fundamentally to relationship satisfaction in the long run (Mazzuca et al., 2019), and in an educational context, it appears to be related to teacher enthusiasm in teacher-student classroom interaction (Frenzel et al., 2018, 2024). Taken together, these findings highlight the importance of research on emotional contagion in social interaction and the need to employ different methodologies to assess different emotion modalities. In the present study, we focus on the participants’ subjective emotional experiences and their facial expressions as one important and visible channel of emotion expression in dyadic social interaction.

### Emotional contagion in dyadic online video conferences

Online video conferences can be defined as the combined transmission of both video and audio signals for the purpose of instant and synchronous communication between two or more physically distant locations (Al-Samarraie, 2019; Christen et al., 2019; Denstadli et al., 2012; Dudding, 2009; Ferran and Watts, 2008; Parasian and Yuliati, 2020; Senft, 2019). Although previous findings stress the importance of research on social interaction in online video conference settings, research on emotional contagion in this context is scarce and, so far, only two studies have investigated this process in a dyadic online video conference setting.

Mui et al. (2018) investigated emotional contagion and reciprocal imitation of smiles in a socially interactive cooperation task (i.e., choosing a target figure from a number of figures based on the descriptions by another person). In this experimental study, the participants (*N* = 52 students) interacted either with a confederate showing positive facial expressions (i.e., the smiling condition) or maintaining a neutral expression (i.e., the neutral condition) via an online video conference system. Importantly, the instructions and reactions of the confederate were videotaped prior to data collection, and all participants, thus, interacted with the same pre-recorded video files instead of authentic real-time interactions with another human being. The participants’ facial expressions were analyzed using automated software and subsequently averaged across the interaction trials. Additionally, they provided self-reports of positive and negative affect before and after the interaction. Based on their analyses (ANOVAs), they found more positive and less neutral facial expressions in the smiling condition, but they did not find convincing evidence for emotional contagion based on the participants’ self-reported affect.

In contrast, Gvirts et al. (2023) investigated interpersonal motor synchrony and emotional alignment in dyadic online video conferences. They brought *N* = 196 students together to dyadically discuss their challenges and difficulties during the COVID-19 pandemic via online video conferences conducted from home. The participants had 2 min to introduce themselves before a five-minute discussion, and they provided self-reports of positive and negative affect before and after the interaction. A research assistant was present in the video conference (with their microphone and camera turned off during the interaction sequences) to start and stop the recordings and to instruct the participants. To quantify the participants’ (upper) body movements and their temporal synchronization, the interaction sequences were videotaped and analyzed using motion energy analysis. The authors concluded from the self-report data that negative affect was transmitted across the interaction partners, but not positive affect, providing first partial evidence for emotional contagion via online video conference systems. Further, based on comparisons of the real movement data with randomly created surrogate data, they found evidence for above chance motor synchrony between the interaction partners in dyadic online video conferences within a time lag of ±5 s.

Taken together, previous findings on the existence of emotional contagion in dyadic online video conferences seem to be inconclusive as of yet, and the existing studies show several important deficits: First, in the study by Mui et al., a pre-recorded confederate was used in the cooperation task instead of naturally interacting individuals. Additionally, this confederate artificially displayed either positive or neutral facial expressions during the video recorded sequences (depending on the experimental condition) instead of facial expressions that stem from their own subjectively experienced emotions. Further, while they aimed to examine the temporally fine-grained and highly dynamic process of temporal coordination and imitation of facial expressions, they did not analyze the participants’ facial expressions on a micro-analytic frame-by-frame level. Instead, they averaged an individual’s facial expression scores across several seconds, thus foregoing the analysis of temporal dynamics in the assessed facial expression data. Second, Gvirts et al. only had their participants discuss “challenges and difficulties.” Therefore, they did not systematically vary different emotion conditions, and their online data collection took place remotely with the participants being in their homes and not in a standardized and controlled lab environment. Moreover, any potential effects of the presence of the research assistant also being present during the interaction (even if muted and non-visible) remain unclear. Last, while they employed time series analysis on fine-grained moment-to-moment motion energy data (i.e., upper body), they did not specifically address the temporal coordination of facial expressions.

### The present study

In the present study, we aimed to contribute to this line of research by implementing a specifically designed lab-based experimental setup using synchronized computers in adjacent lab rooms and a standardized interaction paradigm entailing three different interaction conditions that all participants underwent. In each condition, the dyads were talking about recent experiences that made them feel one of the discrete emotions anger (Anger condition), joy (Joy condition), or sadness (Sadness condition). Given that previous research, for a long time, had focused solely on the emotions joy and anger (e.g., Dimberg et al., 2002), we aimed to broaden the scope of the analyses by expanding the selection of emotions. With the chosen emotions anger, joy, and sadness, we follow more recent studies on emotional contagion (e.g., Olszanowski et al., 2020). These three emotions represent different constellations of valence and arousal (i.e., joy: positive valence/high arousal; anger: negative valence/high arousal; sadness: negative valence/low arousal) and they are commonly associated with distinguished facial expressions that can be rather clearly differentiated. Overall, we consider this selection an economic compromise between the goal of exploring different emotions and, at the same time, balancing the participants’ necessary time investment.

While in previous research, emotional contagion has most often been studied either in field studies assessing data in the participants’ authentic and natural living conditions (e.g., at work or in school) or in experimental studies where participants would be exposed to picture/video stimuli instead of authentic social interaction. Field study designs typically result in greater ecological validity, but lower internal validity, while lab-based designs result in greater internal validity, but lower ecological validity. Given this dichotomy and dilemma between internal and ecological validity in lab-based research, we aimed at a compromise in the present study, striving to achieve the greatest possible ecological validity (i.e., having two participants talk to each other about their own authentic experiences instead of exposing single participants to pictures or video clips of facially expressed emotions) while keeping the greatest possible internal validity (i.e., structured interaction, standardized instructions, etc.). Our key idea was that, in each assessment, both interaction partners take turns being either in a listening or a speaking role. In that, the speaker in each interaction represents a living and authentic stimulus for the other individual during the interaction. We viewed it as empirical evidence for emotional contagion in dyadic online interactions if the listeners’ emotions systematically varied according to the condition created by the speakers’ emotions. To obtain a comprehensive understanding of emotional contagion in these dyadic interaction sequences, we incorporated both self-report measures of subjectively experienced discrete emotions and automated facial action coding for the quantification of facial expressions. The face data was recorded continually and synchronously for the two interacting participants to enable the exploration of fine-grained temporal dynamics across the participants’ facially expressed emotions. Prior to any data analysis, we preregistered our analysis plan in an OSF-repository including the following directed confirmatory hypotheses:

Hypothesis 1: An individual’s self-reported discrete emotions (anger, joy, sadness) differ depending on the emotional state of another person when listening and responding to that person in a structured interaction paradigm in dyadic online video conferences. We expect greater levels of self-reported anger in the Anger condition, greater levels of self-reported joy in the Joy condition, and greater levels of self-reported sadness in the Sadness condition.

Hypothesis 2: An individual’s facially expressed emotions (anger, joy, sadness) differ depending on the emotional state of another person when listening and responding to that person in a structured interaction paradigm in dyadic online video conferences. We expect greater levels of facially expressed anger in the Anger condition, greater levels of facially expressed joy in the Joy condition, and greater levels of facially expressed sadness in the Sadness condition.

In addition to these preregistered hypotheses, we strived to answer a second research question regarding the dyadic temporal alignment of the participants’ facial expressions on a micro-level: Do facially expressed anger, joy, and sadness co-occur to an extent beyond what can be statistically considered coincidental (i.e., above chance) based on cross-recurrence quantification analysis (CRQA)? Our reasoning implies that if emotions were indeed transmitted within each dyad, there should be an above-chance temporal co-occurrence of both participants’ facial expressions of the respective emotion (e.g., facial expressions of joy when listening and responding to another person talking about a joyful event; Joy condition).

To explore this, we used CRQA (Coco and Dale, 2014), a non-linear time series analysis approach, to quantify the degree to which a given facial expression (e.g., facial expressions of joy) co-occurred either perfectly simultaneously or within a short time lag between both interaction partners and compared these results with so-called surrogate time series representing shuffled time series containing the same data points in randomly generated order for each individual in each interaction sequence (Wallot and Leonardi, 2018; see Frenzel et al., 2024, for a similar approach to exploring contagion of facially expressed joy between teachers and students in real-life classrooms).

## Materials and methods

### Sample

The initial sample included *N* = 110 subjects that were assigned to *k* = 55 dyads, depending on their availability for the testing sessions. Three dyads had to be excluded because of technical issues, resulting in a final sample of *N* = 104 subjects (*M_age_* = 23.88 years; *SD_age_* = 5.68; 18–43 years) and *k* = 52 dyads, which met the target sample size estimated from *a priori* power analyses (see “Statistical Analysis” section below). Of these participants, 24 identified as male (23.1%), 77 as female (74.0%), and three as diverse (2.9%), respectively, and 92.3% reported German as their native language. As their highest educational qualification, most of them held a high school degree (*n* = 79 individuals/76.0% of all participants), and some had already completed a Bachelor’s (*n* = 13/12.5%) or Master’s degree (*n* = 11/10.6%). All participants were recruited via mailing lists and messages across different online platforms and networks (e.g., the university’s student newsletter and online learning platform). Participation in the study was voluntary, and all subjects signed an informed consent form and a non-disclosure agreement prior to collecting their data. As an incentive for their participation, they were given additional course credit (in German “Versuchspersonenstunde”) or a small monetary compensation (i.e., 15 EUR). Inclusion criteria were being fluent in German, aged between 18 and 45 years, and having no prior affiliation with the study’s content or procedures (e.g., through research internships). Prior to data collection, 97.1% of the participants reported being familiar or very familiar with online video conferences, and 70.2% reported using them regularly or very regularly.

### Technical setup

**Figure 1:**
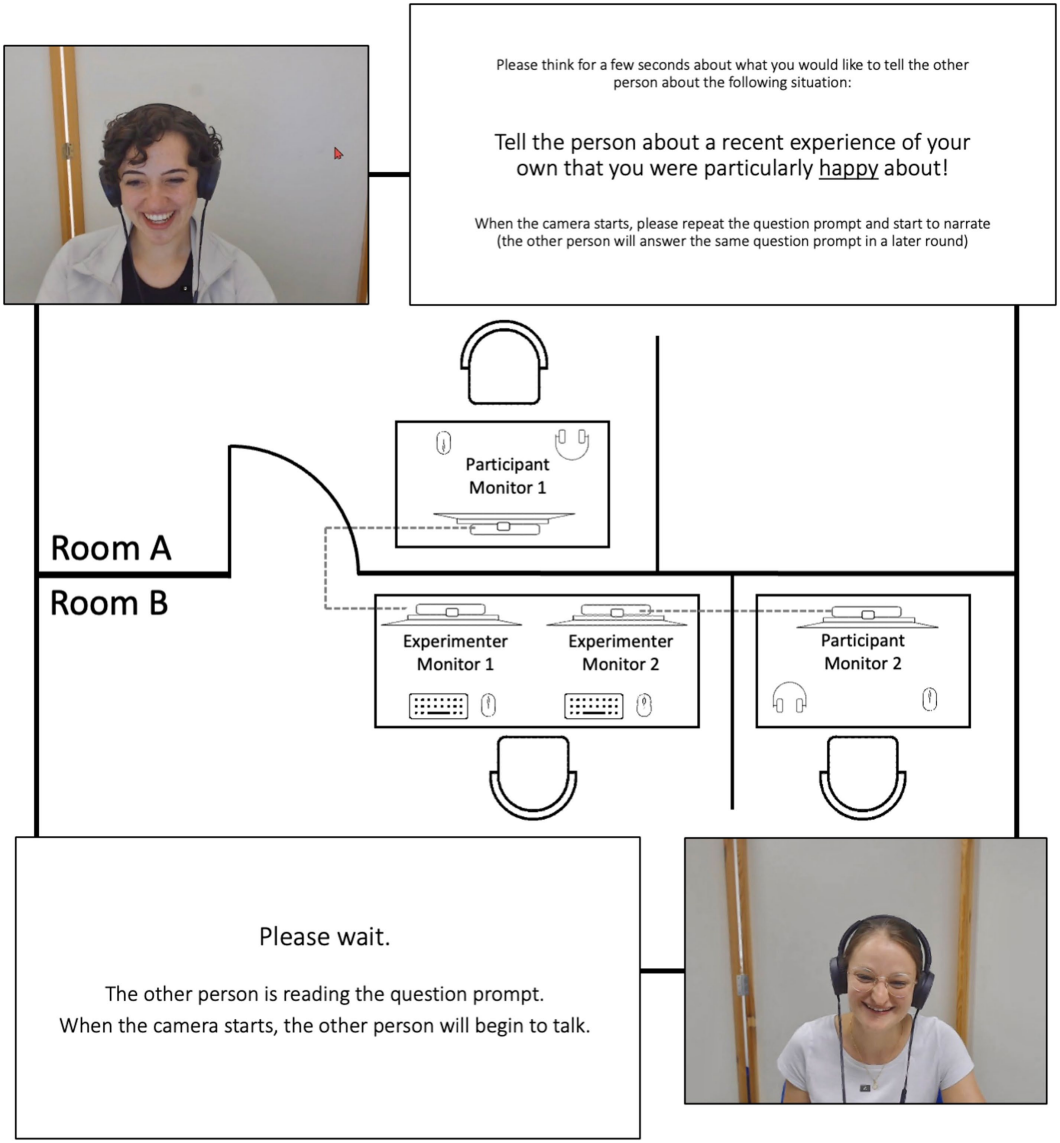
Visual depiction of the study setup in two adjacent lab rooms, exemplary screenshots of two participants, and two exemplary prompt slides. Figure available at https://osf.io/rabv7/ under a CC-BY 4.0 license (pictures shared with participants’ consent).

All data were collected in the Video Lab at the first authors’ institution. During data collection, two computers were used to present the study’s instructions, collect the self-report and facial expression data, and run the online video conference system (see Figure 1 for a visual depiction of our study setup in two adjacent lab rooms). Each computer was connected to two monitors, one for the participants to interact with each other (participant monitors 1 and 2) and one for the experimenter to control the study parameters (experimenter monitors 1 and 2). The two participants were seated individually in adjacent rooms on the same type of cushioned office chair at a desk equipped with the 24″ participant monitor positioned in front of them at approximately 50–70 cm distance and a computer mouse to operate the software during data collection (e.g., to fill in self-report items). In both rooms, mobile partition walls were placed behind the participants to provide a similar neutral background during the online video conference. To transmit the video signal, two webcams with a video resolution of 1,080×1,920 pixels and a frame rate of 30 frames per second (at a sampling rate of 30 Hz) were installed on top of each participant monitor. One webcam was used for the transmission of the video recordings via the online video conference system, and the second webcam was used to record the participant’s face and upper torso for the facial expression analysis. While no audio data was recorded due to data privacy reasons, the audio signal was still transmitted during data collection using small and unobtrusive lavalier microphones and over-ear headphones (same microphone and headphone models for both participants) to enable unobstructed communication between the participants and to shield them from any acoustic noise in the background. The two participants communicated via the video conferencing system “Meet.LRZ”,^1^ which is a highly secure jitsi-based video conferencing service^2^ hosted on official university server systems in Germany, providing typical online video conference features.^3^ Both computers were connected to the same university network directly via LAN cables to ensure an efficient and fast transmission of the video signal. To account for a potential technical transmission lag, we measured the latency at around 100 ms prior to data collection. To automatically integrate and process all self-report and video data and to control stimulus presentation during data collection, the software platform iMotions^4^ was used on both computers.

### Procedure

In each data collection session, two participants were paired up to be tested while interacting with each other via the Meet.LRZ system using an identical technical setup for each of them. Upon arrival, the first participant was immediately placed in front of the participant monitor in room A to avoid contact with the second participant before data collection. Subsequently, the second participant was placed in front of the participant monitor in room B, and both participants were handed out the informed consent form and the non-disclosure agreement to read and sign.

After a short test of the video and audio connection (i.e., a standardized 10-s “Screen Check”) and a subsequent self-report survey assessing basic demographic information, data collection on both computers was started simultaneously. The video conference consisted of separate interaction sequences of up to 2 min. In these sequences, the participants alternately answered a question/instruction prompted to them directly before the sequence or listened/responded to the other participant’s narration. The prompts to the speaking person were either aiming to induce a neutral state (i.e., as the first interaction sequence to get accustomed to the technical setup: “Describe your way to work or university.”) or one of three emotional states (anger, joy, sadness: “Talk about a recent experience of your own that made you particularly angry/happy/sad.”). Both participants answered the three questions alternately. After each interaction sequence, both participants were prompted with the self-report items assessing their emotional states during the previous sequence. Subsequently, they were both presented with a slide only containing the two words “Please wait” on a white background for 5 s. After that, the previously speaking and now listening person was presented with another instruction slide containing only the sentences “Please wait. The other person is reading the question. When the camera is turned on, the other person will begin to talk.” for additional 10 s (see OSF-repository for these instruction slides). The role of the first speaker and listener was randomly assigned to one of the interaction partners. Additionally, we employed two study versions with counterbalanced orders of the emotion conditions.

In both versions, the study started with the Joy condition. Afterwards, in version A, the Anger condition was presented, followed by the Sadness condition. In study version B, the Sadness condition was followed by the Anger condition, respectively. Our reasoning behind this decision was to start the experimental procedure with a positive interaction sequence to help the participants get acquainted to the task of sharing an emotional experience with an unknown interaction partner. In line with this reasoning, we added a less structured interaction sequence (i.e., no assigned speaking or listening roles) comprising a relatively positive instruction for the participants to “cool down” after the experimental procedure and to avoid letting them leave in either an angry or sad emotional state (i.e., unstructured interaction up to 4 min with the following positive instruction: “Tell each other about your greatest passion.”).

For the subsequent analyses, this resulted in six sequences in total: three interaction sequences as the speaking person (i.e., the Anger, Joy, and Sadness condition) and three as the listening/responding person for each participant, respectively. While we limited the maximum duration of each sequence to 2 min based on the piloting of the paradigm, there was no minimum duration prescribed. Once the speaking person was done with their description of the corresponding anger-, joy- or sadness-related experience, they could say “finished” to move on to the next sequence. On average, the participants interacted for 81 s (Min = 15, Max = 120) in the Anger condition, 70 s (Min = 16, Max = 120) in the Joy condition, and 78 s (Min = 22, Max = 120) in the Sadness condition. Immediately after data collection, the participants filled out another short self-report survey before they were handed out a debriefing document with additional information on the study’s goals and objectives.

### Measures

#### Self-reported emotions

To assess the participants’ subjectively experienced anger, joy, and sadness, we used one self-report item for each emotion (three items in total). The participants were asked to indicate how they were feeling (“Please report how you were feeling during the last interaction sequence: How much anger did you feel?/How much joy did you feel?/How much sadness did you feel?”) on a 6-point Likert scale ranging from “none” to “a lot.” The three items were adapted from already existing, well-validated questionnaires (e.g., Frenzel et al., 2016).

#### Facially expressed emotions

In the present study, all video recordings were automatically processed and analyzed using the FACET facial expression classifier within the iMotions software platform (Emotient, 2018). FACET is based on the FACS and represents a commercialized version of the CERT software (Littlewort et al., 2011). In prior validation studies, FACET achieved good to high accuracy scores, especially compared to other algorithms (Dupré et al., 2020; Stöckli et al., 2018). For each participant, 30 video frames per second (sampling rate of 30 Hz) were processed by the analysis software, resulting in an average number of processed frames of *M* = 2,287 per participant (*SD* = 934, Min = 468, Max = 3,599). In our experimental setup, FACET yielded an excellent average rate of recognized and analyzed video frames when the participants were speaking (99.79%) or listening/responding (99.78%).

For each analyzed video frame, the software provides so-called evidence scores for a range of discrete emotions, which represent a logarithmic odds ratio of an expert human coder identifying a facial expression in a given video frame, resulting in time series data of the same length as the respective video recordings. In the present study, we used the scores for the emotions anger, joy, and sadness in correspondence to the three emotion conditions during the online video conference interaction sequences. These scores were first converted into probability values and then dichotomized using a threshold representing a probability of 0.8 or 80% statistical power for detecting the respective facial expression if present in each video frame (see reproducible R scripts for all data processing steps in the online repository available under https://osf.io/rabv7/). In addition to the time series data, we calculated percentage scores for further analyses as the relative share of video frames in which the respective facial expressions of the three emotions under study were coded as present.

### Statistical analysis and data visualization

**Figure 2:**
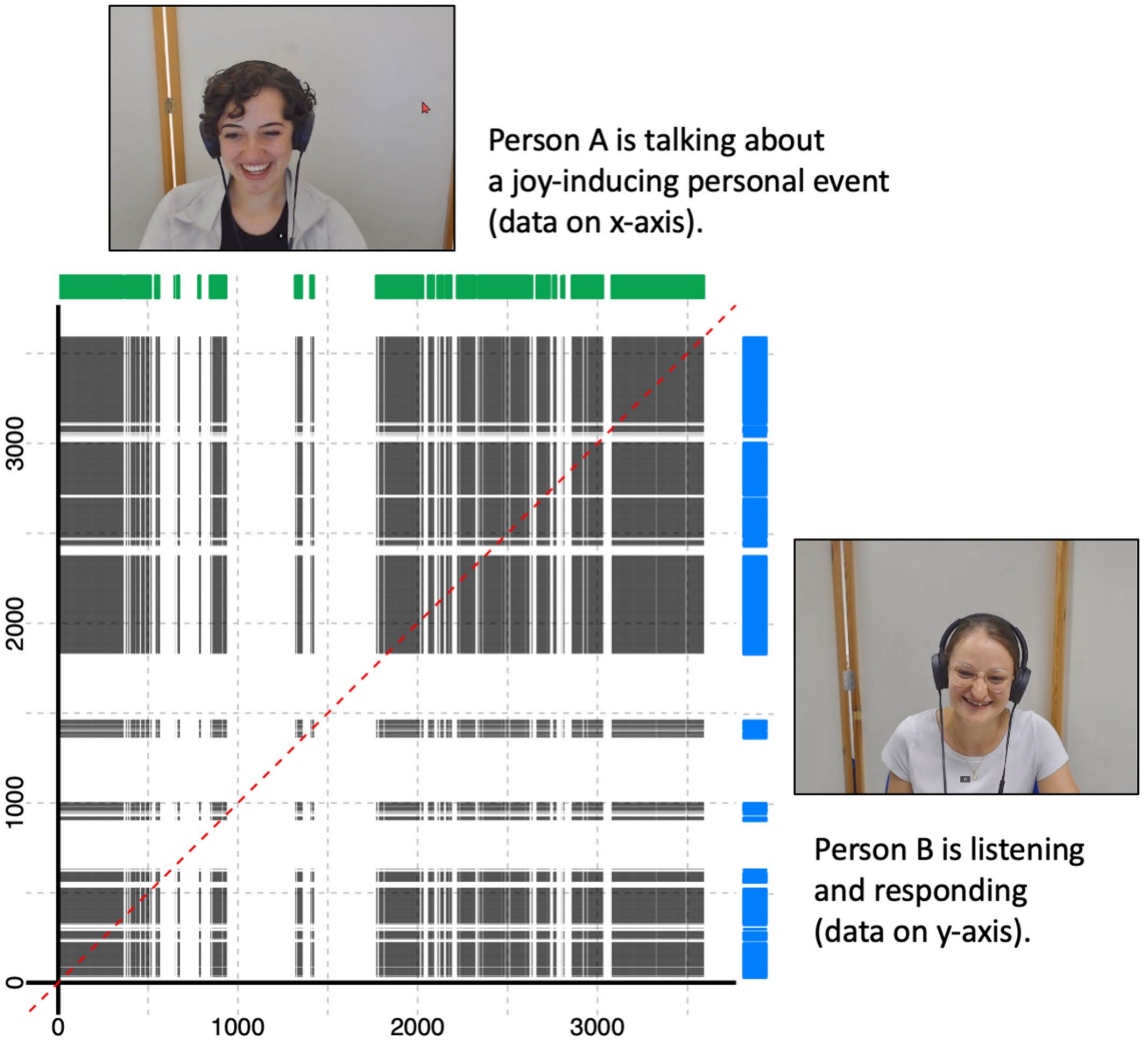
Visual explanation of the cross-recurrence quantification analysis approach involving two facial expression time series (green and blue graphs) corresponding to two exemplary participants. The dotted red line represents perfect temporal co-occurrence. Figure available at https://osf.io/rabv7/ under a CC-BY 4.0 license (pictures shared with participants’ consent).

**Figure 3:**
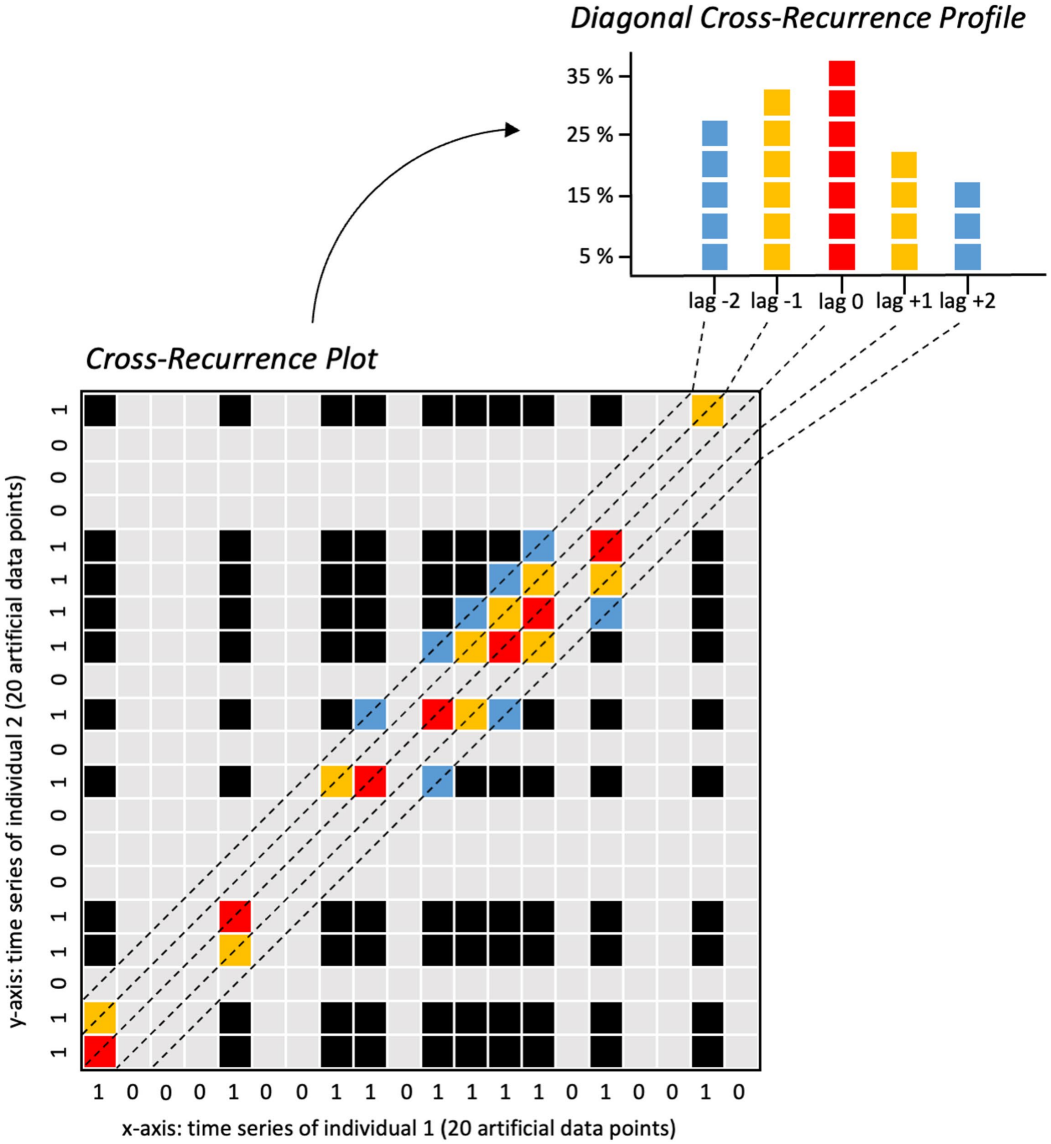
Visual explanation of the diagonal cross-recurrence profile calculation based on two exemplary time series containing artificial data points. Figure available at https://osf.io/rabv7/ under a CC-BY 4.0 license.

All data processing and statistical analyses were performed in R (version 4.3.0), and reproducible scripts have been generated for all reported results and data visualizations (available in the online repository available under https://osf.io/rabv7/). Additionally, Microsoft PowerPoint was used to generate Figures 1, 2 and to assemble Figure 3. Overall, we set the significance level to *p* < 0.05 for all analyses.

#### Preliminary analyses and stimulus check

We conducted preliminary analyses regarding the distribution of the self-reported and facially expressed emotion data. Within the three interaction conditions and in both the speaking and the listening/responding role, none of the data were normally distributed with moderate to very strong kurtosis and skewness values in most of the self-report and, especially, facial expression data (see Tables 1, 2). We tentatively performed three data transformations for all variables (i.e., log-transformation, square-root-transformation, and reciprocal transformation), but none of the transformations resulted in normally distributed data (see Supplementary material S1 and the respective R script in the OSF-repository for a detailed documentation including graphical visualizations and Shapiro–Wilk-Tests). Accordingly, we used non-parametric statistical tests (as preregistered) that do not require normally distributed data.

**Table 1:** Descriptive statistics and data distribution for self-reported joy, anger, and sadness in the respective conditions for the speaking and the listening interaction partner.

Role	Condition	Emotion	*M*	*SD*	Min	Max	Skewness	Kurtosis	Normality
Speaking									
	Anger	Joy	2.63	1.19	1.00	6.00	0.48	2.87	*p* < 0.05
		Anger	2.51	1.66	1.00	5.00	0.44	2.42	*p* < 0.05
		Sadness	1.70	1.00	1.00	5.00	1.32	3.74	*p* < 0.05
	Joy	Joy	4.25	1.22	1.00	6.00	- 0.52	2.94	*p* < 0.05
		Anger	1.13	0.52	1.00	5.00	5.30	35.13	*p* < 0.05
		Sadness	1.18	0.48	1.00	3.00	2.63	9.14	*p* < 0.05
	Sadness	Joy	2.02	0.94	1.00	5.00	0.59	2.70	*p* < 0.05
		Anger	1.53	0.86	1.00	4.00	1.44	3.90	*p* < 0.05
		Sadness	3.06	1.19	1.00	6.00	0.17	2.79	*p* < 0.05
Listening/Responding									
	Anger	Joy	2.71	1.71	1.00	6.00	0.21	2.87	*p* < 0.05
		Anger	1.79	0.98	1.00	5.00	1.05	3.24	*p* < 0.05
		Sadness	1.54	0.93	1.00	5.00	1.97	6.45	*p* < 0.05
	Joy	Joy	4.02	1.19	1.00	6.00	−0.31	3.07	*p* < 0.05
		Anger	1.08	0.39	1.00	4.00	5.81	39.05	*p* < 0.05
		Sadness	1.17	0.43	1.00	3.00	2.44	8.43	*p* < 0.05
	Sadness	Joy	2.06	0.95	1.00	5.00	0.76	3.04	*p* < 0.05
		Anger	1.45	0.85	1.00	5.00	1.99	6.52	*p* < 0.05
		Sadness	2.89	1.11	1.00	6.00	−0.13	2.70	*p* < 0.05

*N* = 104. Response scale: 6-point Likert Scale. We used Shapiro–Wilk tests to test for normal distribution of the data (*p* < 0.05 means that the data is not normally distributed).

**Table 2:** Descriptive statistics and data distribution for facially expressed joy, anger, and sadness in the respective conditions for the speaking and the listening interaction partner.

Role	Condition	Emotion	*M*	*SD*	Min	Max	Skewness	Kurtosis	Normality
Speaking									
	Anger	Joy	62.95	28.11	0	99.94	−0.63	2.24	*p* < 0.05
		Anger	0.72	3.63	0	31.29	6.93	54.07	*p* < 0.05
		Sadness	0.40	1.74	0	13.77	5.77	39.30	*p* < 0.05
	Joy	Joy	63.54	29.89	0	99.83	−0.61	2.14	*p* < 0.05
		Anger	0.61	3.31	0	28.09	7.09	54.47	*p* < 0.05
		Sadness	0.33	1.41	0	10.06	5.73	36.90	*p* < 0.05
	Sadness	Joy	51.34	28.35	0	100.00	−0.17	2.03	*p* < 0.05
		Anger	1.25	6.12	0	43.61	6.07	40.17	*p* < 0.05
		Sadness	0.44	1.77	0	12.28	4.86	27.65	*p* < 0.05
Listening/Responding									
	Anger	Joy	55.78	33.45	0	100.00	−0.19	1.70	*p* < 0.05
		Anger	1.55	9.19	0	89.25	8.67	81.68	*p* < 0.05
		Sadness	0.83	6.76	0	68.71	9.90	99.98	*p* < 0.05
	Joy	Joy	68.01	33.31	0	100.00	−0.88	2.33	*p* < 0.05
		Anger	1.80	9.79	0	75.16	6.13	41.26	*p* < 0.05
		Sadness	0.28	1.70	0	15.39	7.70	64.77	*p* < 0.05
	Sadness	Joy	35.29	31.51	0	100.00	0.70	2.22	*p* < 0.05
		Anger	2.96	13.29	0	82.32	5.12	28.66	*p* < 0.05
		Sadness	0.60	3.12	0	29.23	7.92	71.17	*p* < 0.05

*N* = 104. The reported scores represent facially expressed emotions in % of detected frames. We used Shapiro–Wilk tests to test for normal distribution of the data (*p* < 0.05 means that the data is not normally distributed).

As preregistered, we performed a stimulus check prior to subsequent analyses to test whether we were successful in eliciting subjectively experienced anger, joy, and sadness in the speaking interaction partner during the respective condition. To this end, we calculated Friedman tests (a non-parametric alternative to a repeated-measure ANOVA) applying the Holm *p*-value adjustment method for self-reported anger, joy, and sadness to test for differences between the three conditions for the participant in the speaker role (Holm, 1979). To confirm that the prompts before each sequence were successful in eliciting the respective subjective emotional experiences, we tested whether the participants indeed reported more subjectively experienced anger when talking about an experience that made them angry (Anger condition), more joy when prompted to report about a joyful experience (Joy condition), and more sadness when talking about a sad experience (Sadness condition), compared to the other two conditions, respectively. If the Friedman test was significant, we used pairwise Wilcoxon *post hoc* comparisons to detect differences between these conditions. Again, we employed the Holm *p*-value adjustment method. In addition, we examined the speaking person’s facial expressions of anger, joy, and sadness in all three conditions using the same analysis approach.

#### Preregistered analyses

The following analysis plan was preregistered prior to conducting the analyses. To answer our first research question, we confirmatively tested whether the self-reported (Hypotheses 1) and facially expressed (Hypotheses 2) emotions were transmitted to the listening/responding interaction partner by analyzing differences between the interaction conditions (i.e., the speaker recalls and tells an anger-inducing event, a joy-inducing event, or a sadness-inducing event). We argue that if the self-reported and facially expressed emotions were transmitted from the speaking to the listening/responding interaction partner, the latter should also report and facially express more anger in the Anger condition than in the other two conditions, more joy in the Joy condition, and more sadness in the Sadness condition, respectively. To this end, as preregistered, we again employed Friedman tests with subsequent pairwise Wilcoxon post hoc comparisons applying the Holm *p*-value adjustment method. We used the Holm *p*-value correction to account for multiple testing (i.e., three tests for Hypotheses 1 and three tests for Hypotheses 2) and calculated Cliff’s Delta to quantify effect sizes for the post hoc comparisons (Macbeth et al., 2011). The target sample size was determined based on an *a priori* power analysis using the “WebPower” package in R for 80% statistical power and a large effect size in the Friedman test with three repeated measures (i.e., the three emotion conditions) based on previous findings on emotional contagion and reciprocal imitation of facial expressions using similar analysis approaches (Deng and Hu, 2018; Olszanowski et al., 2020; Wróbel and Olszanowski, 2019) resulting in an estimated sample size of *N* = 100 participants.

#### Additional self-report data analysis: actor-partner interdependence models

To substantiate our preregistered repeated measures analyses of the participants’ self-reported emotions, we additionally applied Actor-Partner Interdependence Models (APIM). APIM offers a dyadic analysis method to account for potential interdependence between two interaction partners in the collected data. This interdependence implies that there may not only be differences between the individuals, but also between the paired dyads (e.g., one dyad experiencing more joy than other dyads). The APIM has shown to be specifically useful when comparing effects of a predictor variable on two unambiguously distinguishable dyad members (Kenny and Kashy, 2014)—which was the case in our design, given the assigned speaking and listening roles. Accordingly, we fit three separate APIMs for self-reported anger, joy, and sadness. In each model, we included the incongruent conditions as two dummy predictor variables each (i.e., the Joy and Sadness conditions for self-reported anger, the Anger and Sadness conditions for self-reported joy, and the Anger and Joy conditions for self-reported sadness) corresponding to an APIM variation using categorical predictors (see Mustanski et al., 2014, for a similar approach). We added both conditions as actor and partner effects and additionally included the covariance between the actor and the partner on self-reported joy. In our case, we defined the listening/responding person as the actor and the speaking person as the partner in all APIM models. One common problem with APIMs is that they are naturally saturated, since they include all possible paths between the predictor and the outcome variables for both members of the dyad (e.g., Kenny et al., 2006). Hence, standard fit indices cannot be used to determine the model’s fit. To account for this issue, we fitted each APIM twice: Once with unrestricted paths and once with restricted effects, where the actor and partner effects were equated (e.g., Kenny and Ledermann, 2010). To compare the fit of the unrestricted and restricted APIM models, we conducted chi-square difference tests. Here, a significant difference indicates that the unrestricted (and, thus, more complex) model shows a significantly better fit over the restricted model and should be preferred. In contrast, a non-significant difference indicates that the models are not significantly different. In this case, for the sake of parsimony, the restricted (and, thus, less complex) model should be preferred. To account for the non-normal distribution in the collected data, the standard errors were kept robust in all models. A reproducible R script for these additional analyses has been uploaded to the study’s OSF repository. Similar to the Friedman analyses, we considered the results indicative of emotional contagion if both the speaking and the listening interaction partner reported significantly more anger in the Anger condition than in the other two conditions, more joy in the Joy condition, and more sadness in the Sadness condition, respectively. Additionally, all three APIM models allowed the interpretation of the covariances between the two interaction partners on the respective emotion. This covariance may also serve as a direct measure of the intensity of the emotional transmission.

#### Additional facial expression data analysis: cross-recurrence quantification analyses

To substantiate our preregistered analyses of the participants’ facial expression data and to answer our second (exploratory) research question regarding the dyadic temporal coordination of the participants’ facial expressions, we aimed to go beyond aggregated percentage scores and make use of the moment-to-moment nature of the assessed time series data. To this end, we used categorical CRQA to quantify the amount of cross-recurrence (i.e., cross-recurrence rate) of facially expressed anger, joy, and sadness for each dyad in the respective interaction condition (i.e., the speaking person recalls and tells an anger-inducing, a joy-inducing, or a sadness-inducing event). CRQA is used to investigate temporal patterns of co-occurrence between two time series (see Figure 2 for a visualization). It allows quantifying the amount of co-occurrence and/or lagged co-recurrence (i.e., the cross-recurrence rate) for a defined variable of interest for two interacting persons (Fusaroli et al., 2014; Wallot and Leonardi, 2018), which makes it a highly suitable analysis approach to quantify the degree of temporal coordination of facial expressions between two individuals.

To calculate the cross-recurrence rates of facially expressed anger, joy, and sadness at specific time lags within an *a priori* defined time window, we used the R function “drpfromts()” from the R package “crqa” to calculate so-called diagonal cross-recurrence profiles (Coco and Dale, 2014) for each dyad and interaction sequence. We applied a time window of ±5 s consisting of ±150 video frames based on previous literature on temporally coordinated behaviors in dyadic interaction (e.g., Gvirts et al., 2023; Paulick et al., 2017; Schoenherr et al., 2019). Within this time window, the cross-recurrence rate for each time lag from −150 to +150 video frames (incl. lag 0 = 301 time lags overall) was calculated (see Figure 3 for a visualization).

To test whether the calculated cross-recurrence rates significantly differed from random cross-recurrence rates that solely originate from chance, we used a surrogate data approach. Surrogate time series are created by randomly shuffling the time series of each interaction partner in the dyad using the sample() function in R (Wallot and Leonardi, 2018), thereby removing the systematic temporal dynamic and coordination originating from the dyadic interaction of the two individuals, but at the same time perpetuating the event frequencies of the corresponding facial expressions across each real vs. surrogate time series. Consequently, the surrogate time series serve to estimate the amount of cross-recurrence for the facial expressions of interest as could be expected by chance (see Supplementary material S2 for a visualization of the real versus the shuffled surrogate time series in two exemplary interaction sequences). Subsequently, we averaged the cross-recurrence rates (i.e., percentage scores) across all individuals in both the real and the surrogate data at each time lag and conducted pairwise comparisons (Wilcoxon paired signed-rank test with an effect size *r* and the Holm *p*-value adjustment method; Field et al., 2012), for the 301 lags of interest (−150 to +150 lags). The results of these pairwise comparisons indicate whether the original cross-recurrence rates for facially expressed anger, joy, and sadness in the respective conditions were, significantly different from the surrogate cross-recurrence rates in this lag window of interest.

## Results

### Preliminary analyses

#### Descriptive statistics

All descriptive statistics for the participants’ self-reported emotions are summarized in Table 1 and for facially expressed emotions in Table 2, respectively. Regarding their self-reported *subjectively experienced emotions*, the participants, when speaking and listening, reported higher levels of joy overall and comparatively lower levels of anger and sadness. Regarding their facially expressed emotions, the participants, when speaking and listening, displayed only very little anger and sadness across all conditions (less than 3% of the total time speaking or listening). In contrast, joy was facially expressed very frequently—more than half of the time speaking or listening—in all three conditions and both roles. When further comparing the two roles during the interaction, the participants displayed greater levels of facially expressed joy across all three conditions in the speaking role (*M* = 59.28%) as compared to the listening role (*M* = 53.03%), but less facially expressed anger and sadness in the speaking role (anger: *M* = 0.86%; sadness: *M* = 0.39%) versus the listening role (anger: *M* = 2.10%; sadness: *M* = 0.57%), respectively. However, there were no significant differences between the speaking and the listening role across all conditions when tested using non-parametric pairwise comparisons (anger: *p* = 0.192; joy: *p* = 0.170; sadness: *p* = 0.070; *p*-values adjusted using the Holm-correction method).

#### Stimulus check

Regarding the *subjectively experienced emotions* of the speaking person during the interaction sequences, the overall Friedman tests indicated significant differences in self-reported anger between all three conditions (*χ*^2^(2) = 112.07, *p* < 0.001), with the greatest levels of anger in the Anger condition as revealed by post-hoc analyses. Similarly, subjectively experienced joy (*χ*^2^(2) = 148.48, *p* < 0.001) as well as sadness (*χ*^2^(2) = 135.35, *p* < 0.001) differed significantly between all three conditions, with greatest levels of self-reported joy in the Joy condition and self-reported sadness in the Sadness condition, respectively. Regarding the *facially expressed emotions* of the speaking person during the interaction sequences, the overall Friedman tests indicated no significant differences in facially expressed anger (*χ*^2^(2) = 1.32, *p* = 0.605) nor sadness (*χ*^2^(2) = 2.39, *p* = 0.605) between all three conditions. Facially expressed joy, on the other hand, differed significantly between the three conditions (*χ*^2^(2) = 57.81, *p* < 0.001). According to the post-hoc tests, expressed joy was significantly lower in the Sadness condition than in the Joy and Anger condition, but the latter two conditions were not significantly different.

Overall, we conclude that we were successful in eliciting the intended subjective emotional experiences in the speaking person (i.e., anger, joy, and sadness) during the respective condition, which acts as the basis of our interactional paradigm and all subsequent analyses. However, the participants’ subjective emotional experiences were not fully congruent with the expressions on their faces during the interaction, which is in line with previous findings on the (in)congruence of self-reported and facially expressed emotions in authentic social interaction (Barrett et al., 2019; Keltner et al., 2019).

### Preregistered analyses

#### Hypothesis 1: self-reported emotions are transmitted in dyadic online video conferences

For self-reported anger in the listening role, the overall Friedman test was significant (*χ*^2^(2) = 53.10, *p* < 0.001), with significantly more self-reported anger in the Anger condition as compared both to the Joy condition (medium effect of *δ* = 0.43, *p* < 0.001) and to the Sadness condition (small effect of *δ* = 0.21, *p* < 0.001) as revealed by *post hoc* tests. For self-reported joy, the overall Friedman test was also significant (*χ*^2^(2) = 128.94, *p* < 0.001), and post hoc tests showed significant differences between all three pairwise comparisons. There were large effect sizes with more joy in the Joy condition as compared both to the Anger condition (*δ* = 0.57, *p* < 0.001) and to the Sadness condition (*δ* = 0.78, *p* < 0.001). For self-reported sadness in the listening role, the overall Friedman test was also significant (*χ*^2^(2) = 140.22, *p* < 0.001). Again, there was significantly more self-reported sadness in the Sadness condition as compared both to the Joy condition (*δ* = 0.79, *p* < 0.001) and to the Anger condition (*δ* = 0.63, *p* < 0.001), with large effect sizes for both comparisons. In sum, the listening individuals reported significantly greater levels of subjectively experienced anger, joy, and sadness when interacting with a person talking about a personally relevant anger-/joy-/sadness-inducing event. Thus, subjectively experienced anger, joy, and sadness appear to be transmitted in dyadic online video conferences.

#### Hypothesis 2: facially expressed emotions are transmitted in dyadic online video conferences

For facially expressed anger in the listening role, the overall Friedman test was significant (*χ*^2^(2) = 11.02, *p* = 0.008). However, the pairwise comparisons revealed that listeners did not express anger significantly more frequently in the Anger as compared to the Joy condition (*p* = 0.085) and not significantly more often in the Anger condition than in the Sadness condition (*p* = 0.577). For facially expressed joy, the overall Friedman test revealed significant differences between the three emotion conditions (*χ*^2^(2) = 74.08, *p* < 0.001). *Post hoc* pairwise Wilcoxon tests showed significantly more facially expressed joy in the Joy condition as compared both to the Anger condition (small effect of *δ* = 0.22, *p* < 0.001) and to the Sadness condition (large effect of *δ* = 0.51, *p* < 0.001). For facially expressed sadness, the overall Friedman test did not yield significant differences between the three conditions (*χ*^2^(2) = 5.65, *p* = 0.594). In sum, individuals displayed facial expressions of joy in the Joy condition more frequently than when listening and responding to a person who spoke about an anger- or sadness-inducing event, providing further support for the transmission of joy during video conferences. However, the evidence for more frequent facial expressions of anger or sadness when listening and responding to someone talking about an anger- or sadness-inducing event, respectively, was weak.

### Additional self-report data analyses: actor-partner interdependence models

For self-reported anger, the unrestricted model with different parameters for the listening versus speaking person had a significantly better fit to our data than the restricted model (∆*χ*^2^(∆df2) = 27.813, *p* < 0.001). We, thus, selected this model over the restricted model. There was a small significant covariance between the listening and the speaking person’s self-reported anger (*β* = 0.17, *p* < 0.001). In line with expectations, participants reported most anger in the reference condition of Anger; this applied to both the listening (effect of Joy condition *β* = −0.40, *p* < 0.001; effect of Sadness condition *β* = −0.19, *p* < 0.001) and the speaking interaction partner (effect of Joy condition *β* = −0.61, *p* < 0.001; effect of Sadness condition *β* = −0.40, *p* < 0.001). These effects of condition appeared to be stronger for the speakers than for the listeners.

For self-reported joy, the chi-square difference test indicated no significant difference between the restricted and the unrestricted model (∆*χ*^2^(∆df2) = 3.14, *p* = 0.21). Thus, we selected the more parsimonious restricted model for its simplicity, suggesting the actor and partner effects can be equated without significantly worsening the model fit. The results indicated a small- to medium-sized significant covariance between the listening and speaking person’s joy (*β* = 0.25, *p* < 0.001). Both the Anger condition and the Sadness condition had strong negative effects on the listeners’ (Anger: *β* = −0.49, *p* < 0.001; Sadness: *β* = −0.70, *p* < 0.001) and the speakers’ (Anger: *β* = −0.48, *p* < 0.001; Sadness: *β* = −0.69, *p* < 0.001) self-reported joy.

For self-reported sadness, the chi-square difference test indicated no significant difference between the restricted and the unrestricted model and, thus, the more parsimonious restricted model was, again, preferred for its simplicity (∆*χ*^2^(∆df2) = 1.76, *p* = 0.40). There was a medium-sized significant covariance between the listening and speaking person’s sadness (*β* = 0.43, *p* < 0.001). The Anger condition had strong negative effects on both the listeners’ (*β* = −0.55, *p* < 0.001) and the speakers’ (*β* = −0.53, *p* < 0.001) self-reported sadness. The Joy condition yielded similar results with even larger effects on both the listeners’ (*β* = −0.73, *p* < 0.001) and speakers’ (*β* = −0.70, *p* < 0.001) self-reported sadness. Additional graphical visualizations of a detailed description of all models and their parameters are provided in Supplementary material S3.

### Additional facial expression data analyses: cross-recurrence quantification analysis

Regarding our second research question (i.e., above-chance cross-recurrence), the descriptive statistics of the cross-recurrence rates aggregated across all time lags during the time window of interest (i.e., from −150 to +150 lags) for the real versus the shuffled surrogate data are depicted in Table 3. Regarding facially expressed anger in the Anger condition, the average cross-recurrence rates at each time lag in the real data were very low (*M* = 0.007%). In other words, the relative share of data points (i.e., video frames) in which both interaction partners displayed facial expressions of anger either simultaneously or within a lag of ±5 s was very small. Nevertheless, pairwise comparisons with the surrogate data (*M* = 0.004%) revealed that the cross-recurrence rates in the real dyads were significantly larger than the cross-recurrence rates in the respective surrogate data (*p* < 0.001, *r* = 0.75 which represents a large effect; Field et al., 2012).

**Table 3:** Descriptive statistics of the cross-recurrence rates in the real versus the shuffled surrogate data across all time lags.

Data	Condition	Emotion	*M*	*Mdn*	SD	Min	Max
Original							
	Anger	Anger	0.007	0.006	0.003	0.002	0.013
	Joy	Joy	47.5	47.5	0.65	46.4	48.5
	Sadness	Sadness	0.006	0.006	0.003	0.001	0.014
Surrogate							
	Anger	Anger	0.004	0.004	0.001	0.001	0.007
	Joy	Joy	46.6	46.6	0.04	46.5	46.7
	Sadness	Sadness	0.010	0.010	0.002	0.006	0.016

*N = 301 time lags. The reported cross-recurrence rates are depicted in % averaged across participants at each time lag.*

For facially expressed joy in the Joy condition, in contrast, the average cross-recurrence rates during the time window of interest in the real data were high (*M* = 47.5%). That means that both interaction partners displayed facial expressions of joy either simultaneously or within a lag of ±5 s in almost half of the time of their interaction. Subsequent pairwise comparisons with the surrogate data (*M* = 46.6%) revealed that the real cross-recurrence rates were significantly above chance; with significantly higher levels of cross-recurrence in the real data than in the surrogate data (*p* < 0.001, *r* = 0.81; large effect).

Regarding facially expressed sadness in the Sadness condition, the average cross-recurrence at each time lag, again, was again very low (*M* = 0.006%). Moreover, in this condition, it turned out that the cross-recurrence rates were in fact higher in the surrogate data (*M* = 0.010%) than in the real data (*M* = 0.006%), and this difference was significant (*p* < 0.001, *r* = 0.76; large effect). This implies that if one interaction partner facially displayed sadness, the probability for the other interaction partner simultaneously or within a lag of ±5 s was significantly lower than could be expected by chance.

## Discussion

Existing evidence for emotional contagion in dyadic face-to-face interaction is scarce, but their relevance for important socio-emotional outcomes, and the ubiquitous use of online video conference applications in everyday social interaction in today’s world is undisputed. The overarching goal of the present work was to investigate the existence of emotional contagion based on self-report and facial expression data in dyadic social interaction via an online video conference application. To this end, we implemented a specifically designed experimental setup involving synchronized computers and a structured interaction paradigm, bringing two interaction partners alternately into a speaking and a listening/responding role while systematically manipulating the emotional context the speaking person was prompted to address (i.e., Anger, Joy, and Sadness conditions). In this setup, we assessed both self-reported subjective experiences and facial expressions of emotions of both individuals. To assess whether emotional contagion happens in such dyadic online video interaction (research question 1), we tested whether the listening/responding persons would self-report and facially express the respective emotion corresponding to the emotional context the speaking person was prompted to (i.e., anger, joy, sadness). To further explore whether such emotional contagion is rooted in a fine-grained micro-level temporal coordination of the two interaction partners’ facial expressions of the condition-specific emotions, we additionally applied CRQA. This nonlinear time series analysis approach allowed us to quantify the degree to which a given facial expression (e.g., facial expressions of joy) co-occurred either perfectly simultaneously or within a lag of ±5 s among both interaction partners.

### Emotions are transmitted in dyadic online video conferences

When comparing the participants’ self-reported emotions across the three emotion conditions, our analyses provided clear evidence that the listening/responding individuals reported greater levels of subjectively experienced anger, joy, and sadness in the respective emotion conditions. More specifically, they reported greater levels of subjectively experienced anger when listening/responding to their interaction partner’s report of a recent experience that made them particularly angry (Anger condition) compared to a joyful (Joy condition) or sad experience (Sadness condition). Analogically, the participants reported the highest joy levels when listening to someone reporting an experience that made them happy (Joy condition) and most sadness when listening to someone elaborating on something that made them particularly sad (Sadness condition). These results were corroborated by the dyadic APIM analyses; hence these result patterns also hold when accounting for the interdependence in dyadic data. Furthermore, besides the replication of the differences between the conditions, the significant covariances between the actor and partner self-reported anger, joy, and sadness provided further evidence for emotional contagion of each of these three emotions as operationalized through self-report. Overall, these findings add to the inconclusive results from the studies conducted by Gvirts et al. (2023) and Mui et al. (2018). While our conclusion is in line with the findings by Gvirts et al. (2023), who recently reported a similar pattern when examining the transmission of positive and negative affect via an online video conference software, it contradicts the results by Mui et al. (2018). Mui et al. had examined the participants’ self-reported emotional reactions to pre-recorded videotapes of confederates and did not find evidence for emotional contagion in video conferences. Thus, authentic, real-time encounters between rather naturally interacting communication partners seem to be a necessary condition for emotional contagion to take place in dyadic online video conferences.

While the evidence for emotional contagion as operationalized through self-report data was compelling, our findings regarding facially expressed emotions were mixed. On the one hand, we found that participants in the listening/responding role showed the highest levels of facially expressed joy in the Joy condition as opposed to both the Anger and Sadness condition. On the other hand, we found no evidence for higher frequencies of facially expressed anger and sadness in the respective emotion condition. Importantly, however, the three emotions differed greatly in the frequencies of their respective facial expressions. As detailed in Table 2, joy was frequently expressed in both roles and all three emotion conditions with mean values ranging from 35.29 to 68.01% of the video frames. In contrast, anger and sadness showed very low frequencies, respectively, with mean values ranging from 0.61 to 2.96% for facially expressed anger and from 0.28 to 0.83% for facially expressed sadness and the majority of data points being close to zero.

On the one hand, these differences in the observed frequencies of facially expressed joy as opposed to facially expressed anger and sadness in our data seem to be a somewhat surprising finding. Given the substantial self-reported levels of anger and sadness, expecting similarly substantial levels of facially expressed anger and sadness would be in line with classic emotion theories, such as Basic Emotion Theory (e.g., Keltner et al., 2019), proposing relatively high congruency between subjective experiences and facial expressions of emotions when expressed freely (see also Matsumoto et al., 2008). On the other hand, the reduced levels of observed facial expressions of anger and sadness during the interaction sequences could be seen as support for similarly prominent propositions that facial expressions may not necessarily align with an individual’s subjective emotional experience, especially in relatively authentic and naturalistic social interaction. In such contexts, research has shown that potential social expectations toward what could be seen as appropriate behavior in a particular situation as well as culturally shaped display rules might constrain an individual’s expressiveness (Barrett et al., 2019; Matsumoto and Hwang, 2013). In line with this view, especially facial displays of negative or aversive emotions, such as anger and sadness, are thought to be likely suppressed or masked during the interaction to, for example, regulate one’s own or the other individual’s emotional experiences (e.g., Gross and John, 2003; see also Petrova and Gross, 2023).

Taken together, our findings support that interaction partners converge in their subjectively experienced anger, joy, and sadness during online conversations as well as temporally align their facial expressions of joy. However, the face does not seem to be an important channel for transmitting anger and sadness during online conversations. These non-significant findings for facially expressed anger and sadness need to be corroborated in future replication studies, given that only large effects could be detected with sufficient statistical power and potential effects for anger and sadness could be smaller than the reported effect sizes in previous research.

### Above chance cross-recurrence of facially expressed emotions

To answer our second research question, we went beyond aggregated percentage scores and repeated-measures analyses and, instead, made use of the dyadic and highly dynamic moment-to-moment nature of the assessed facial expression data. To this end, we applied non-linear time series analyses (i.e., CRQA) to the dyadic time series data in combination with pairwise comparisons with so-called surrogate time series comprising the same data points, but in a randomly shuffled order. Regarding facially expressed anger and joy, the real cross-recurrence rates within the pre-defined time window of ±5 s were significantly larger than the surrogate cross-recurrence rates at each time lag in the Anger condition and in the Joy condition, respectively. This finding implies that, when listening and responding to a person talking about a recent personally relevant event that made the speaking person particularly joyful or angry, facial expressions corresponding with those of the speaker appeared to be imitated in systematic temporal alignment. In contrast, the real cross-recurrence rates of facially expressed sadness were significantly lower than the surrogate cross-recurrence rates at each time lag in the Sadness condition. In other words, when listening and responding to a person talking about a recent personally relevant event that made the speaking person particularly sad, facially expressed sadness seemed to co-occur temporally less frequently than as expected by chance. However, it is important to note that only facially expressed joy showed substantially large cross-recurrence rates, whereas the cross-recurrence rates of facially expressed anger and sadness were extremely low with many data points close to zero. More specifically, facially expressed anger and sadness co-occurred in both interaction partners in less than 0.01% of all possible data points within the time window of ±5 s and facially expressed joy co-occurred in almost 50% of all data points, respectively. Correspondingly, the CRQA findings for facially expressed anger and sadness seem to be largely driven by interaction sequences in which the CRQA resulted in no or almost no temporal co-occurrence between the two interacting individuals (which is not surprising, given the overall very low individual frequencies of facially expressed anger and sadness).

Hence, we propose to interpret the CRQA findings regarding facially expressed anger and sadness as rather descriptive information on the few dyads exhibiting relatively substantial levels of cross-recurrence. In contrast, we consider our CRQA findings for facially expressed joy as robust evidence for emotional contagion and the temporal interpersonal coordination of facially expressed joy in dyadic online video conferences. On the one hand, this interpretation aligns with the findings by Mui et al. (2018), who found first evidence for smile mimicry in online video conferences using aggregated facial expression data. On the other hand, it is important to note that our findings, thus, cannot be generalized to emotional contagion overall, but instead only apply to facial expressions of joy. Herein, the results contribute to research on joy transmission by analyzing the cross-recurrence patterns of facial expressions of joy in extensive moment-to-moment time series data among naturally interacting individuals.

### Limitations

When interpreting the reported findings, several limitations should be taken into account. First, while we reached our target sample size based on our a-priori power analyses, the sample’s composition was rather homogeneous, which might decrease the generalizability of our findings. The majority of the participants were students at an academic institution, and accordingly, their mean age was rather young, and they were predominantly well-educated. This demographic skew in our data could lead to biased conclusions in that older individuals may have lower levels of familiarity and proficiency with online video conference software (Parasian and Yuliati, 2020), and their experienced emotions and facial expressions when using video conference systems may be influenced by other factors outside of the actual social interaction. However, given that especially universities and post-graduate schools represent interaction contexts in which online video conference tools will remain implemented regularly in the future (Bader et al., 2022), students represent a population that is of particular interest for research on emotional experiences in online video conference settings.

Second, in the present study, we only assessed data in the context of online video conferences but not in the context of face-to-face interactions. While our analyses provide convincing evidence pointing toward the existence of emotional contagion in videoconference contexts, they do not allow for any inferences regarding potential level differences of the amount of emotional contagion across face-to-face as contrasted with video conference settings. The present study’s experimental setup, technical procedures, and novel interactional paradigm, however, can be used as a starting point for future research involving direct replications of our findings as well as conceptual adaptations, including a direct comparison across dyadic face-to-face interactions and video-based online interactions.

Third, when randomizing the order of the emotion conditions presented to the participants, we chose to start both study versions with the Joy condition, followed by either the Anger condition (study version A) or the Sadness condition (study version B) in a counterbalanced order. Our reasoning behind this was to give all participants the same positive start to the interaction task and to make it easier for all participants to familiarize themselves with the task of sharing an emotional experience with an unknown interaction partner. Hence, our findings are limited to this order (positive first, then negative), and it may well be that participants might have been prone toward a more positive interpretation of the following emotion conditions, which is possibly reflected in the less frequent occurrence of facially expressed anger/sadness in the data collected.

Fourth, on a methodological level, using facial expressions as indicators of emotional experiences, and specifically, applying automated facial expression analysis algorithms is not without criticism (Barrett et al., 2019; Cross et al., 2023). On the one hand, there is an ongoing debate around the congruence of facial expressions with the underlying emotional experiences. Regarding this issue, we do not propose that subjective emotional states can be inferred directly from an individual’s facial expressions. Instead, we see the human face as visual communication channel in interpersonal interaction. In this sense, we used automated facial expression analysis to measure changes in the outward appearance of an individual’s face, while not necessarily coinciding with an individual’s subjective experiences, to explore the degree to which those expressions are transmitted even across the “obstacle” of a digitally mediated social interaction. On the other hand, regarding the issue of validity and reliability of automatic facial expression analysis, there are several important aspects to discuss. While recent validation studies (see Dupré et al., 2020 for an overview) reported that automatic facial expression analysis algorithms offer sufficiently high reliability in comparison to human FACS coders (Höfling et al., 2022; Küntzler et al., 2021; Skiendziel et al., 2019; Stöckli et al., 2018) and to itself when measured at two measurement points (Borsos et al., 2022), the reported results may have been influenced by deficiencies in the facial expression analysis software that we used to process the video data (i.e., FACET). Facial expression analysis algorithms are usually trained and validated using image and video databases that contain posed and/or spontaneous facial expressions. In published validation studies, such algorithms typically achieve lower accuracy scores for spontaneously displayed and dynamic facial expressions as for deliberately posed and static expressions. Given that the participants in our experimental setup engaged in rather authentic and naturalistic social interactions, including mouth/lip movements resulting from speaking, insufficient accuracy of the FACET classifier for such authentic and naturalistic facial expressions could have contributed to the low detection rates for anger and sadness in the present work. However, when comparing the frequencies of facially expressed emotions between the speaking and the listening role (see Table 2), the participants in both roles showed substantial levels of facially expressed joy and comparably low levels of both facially expressed anger and sadness, respectively, but there were no significant differences between the obtained levels of facially expressed emotions between the two roles. In our view, this indicates that there is no systematic bias in the recognized facial expressions solely arising from being either speaking or listening during the interaction. Moreover, the FACET classifier in particular has been repeatedly reported to outperform other facial expression analysis algorithms (Stöckli et al., 2018; Dupré et al., 2020) and to achieve similar performance rates as other measures of facial expressions/muscle movement (Beringer et al., 2019). Hence, we conclude that facial expressions—independent of the underlying emotional state—represent highly relevant nonverbal cues and a channel for the interpersonal communication of subjective emotional experiences that can be assessed using automated facial expression analysis.

Fifth, self-report measures are inherently susceptible to demand characteristics (Nichols and Maner, 2008), meaning that participants’ responses may be influenced by perceived experimenter expectations or social desirability. In our study, one potential concern is that the self-report items closely resembled the prompts the speaking interaction partner received before their interaction. Therefore, participants could have been prone toward reporting higher values on those items that were congruent with the emotion they were prompted to talk about. However, in our paradigm, several factors mitigated this concern: (1) Due to the interaction after the prompt, there was a temporal gap between the initial prompt and the moment the speaking partner provided the self-reports, reducing the likelihood of immediate response bias; (2) we observed consistent emotional contagion effects not only in the speaking interaction partner, but also in the listening partner—who did not receive a prompt before answering the self-report items; (3) the participants were unaware of the study’s specific hypotheses, further reducing the risk of systematic demand effects. Still, future replications should consider rewording the prompts to elicit the target emotions more implicitly or employing more nuanced self-report measures.

Last, in contrast to most previous research on emotional contagion, and to address the internal-ecological validity trade-off dilemma, the present study aimed at a compromise between internal validity and ecological validity by having real persons talk to each other about their own authentic experiences in a best-possibly structured interaction with standardized instructions, technical equipment, and situational circumstances in the lab. Resulting from this decision, we cannot completely rule out potential influences of other processes that are either related to emotional contagion (e.g., empathy facets like prosocial reaction tendencies, sympathy, perspective taking) or typically prevalent in authentic social interaction outside of the lab (e.g., social norms, social desirability, display rules, or expectations about “correct” reactions). Thus, when interpreting our findings, these processes have to be considered as additional factors potentially influencing the participants’ interactions.

### Implications for practice and future research

Given the reported empirical evidence for emotional contagion of self-reported subjectively experienced anger, joy, and sadness in video-based dyadic interaction, several implications for the practical usage of online video conference systems can be derived. On the one hand, online video conference applications seem to be capable of transmitting emotional signals in social interaction. This represents a practically useful finding for any interactional context in which emotions are important; be they private (e.g., family interaction or romantic relationships), or professional. For example, in an educational context, when having online classes, teachers are likely to transmit subjectively experienced joy to their students and vice versa, similar to face-to-face settings (Frenzel et al., 2018). This dynamic and reciprocal process stresses the importance of authentically expressing positive emotions in class, even when teaching in an online video conference setting (Keller et al., 2018; Taxer and Frenzel, 2018; Schwab et al., 2022).

Furthermore, in a clinical setting, online video conference applications are used increasingly to deliver psychotherapy or clinical counseling sessions (e.g., Newbronner et al., 2022). In psychotherapy and counseling, the importance of therapists’ or counselors’ emotional reactivity to their clients has been shown repeatedly in previous research (e.g., Barzilay et al., 2022) and the impact of a psychotherapist’s awareness of the occurring emotional contagion processes on treatment outcomes has been highlighted (Abargil and Tishby, 2022; Nissen-Lie et al., 2022). Here, the present findings provide valuable insights for mental health professionals by highlighting the relevance of interpersonal emotional processes like emotional contagion in dyadic social interaction during online video conference applications. The present study has shown that individuals’ subjective emotional experiences may not fully coincide with their facial expressions and, even though joy was visible in the participants’ faces with substantial frequency, anger and sadness were not. While this finding has to be further replicated in future studies, it suggests that another person’s internal subjective emotional experiences might be difficult to recognize during online video interaction. Given the additional lack of other visible channels for emotional signals (e.g., body movement outside of the camera’s field of view), interacting via online video conference applications in an emotion-aware way might require heightened sensitivity to other—potentially more subtle—emotional signals (e.g., specific voice characteristics or vocabulary).

Based on the experiences during the COVID-19 pandemic, we can most likely assume that online video conferences will remain a standard tool for social interaction in the context of work, healthcare, and our personal lives. But even beyond this recent catastrophic global event, people will have to rely on online video conference applications when other physically more proximal solutions are not available. In the past, this has already been the case either because of large geographical distances (Alnemary et al., 2015; Antoun et al., 2014), restricted individual mobility (Lazzari et al., 2011), or in remote and inaccessible locations (Burton et al., 2016; Gibson et al., 2011; Sørlie et al., 1999). Taken together, research on emotional contagion and related phenomena in online video conference settings appears to be of eminently high practical relevance for both private and professional social interaction in the future. Based on our participants’ feedback and the successful implementation in the present study, we deem our video-based experimental setup a feasible methodological approach suited to collect individuals’ subjective self-report and facial expression data in a reasonably authentic—yet substantially standardized—dyadic online video conference setting.

In light of the present study’s limitations, the reported analyses and findings should be replicated in larger and more diverse samples to corroborate our results. To go beyond emotion-specific aggregated facial expression scores, future research should expand the analyses on the level of specific action units. To this end, a collaborative effort of different laboratories and researchers could help to sufficiently increase sample size and statistical power to investigate facial expressions of emotions and their role in social interaction and interpersonal functioning on a larger scale. In the sense of a so-called multiverse analysis (e.g., Steegen et al., 2016), systematically comparing different facial expression algorithms and data processing strategies and subsequently evaluating their results from a meta-analytical perspective could provide additional insights on facial expressions in social interaction. In addition to that, future research should address more specific and ecologically valid interaction contexts, such as education or psychotherapy, more diverse samples (i.e., recruited outside of the university context), as well as more emotion modalities than self-reported subjective experiences and automated facial expression coding (e.g., facial electromyography, voice characteristics, physiological measures, vocabulary use/sentiment analysis). Moreover, different variables related to emotional contagion should be assessed in addition to the participants’ emotions, such as an individual’s susceptibility to emotional contagion (e.g., Marx et al., 2024), cognitive subfacets of empathy (e.g., perspective-taking, imagining being in the other person’s situation, remembering a similar experience of oneself), or prosocial behavioral reaction tendencies (e.g., the urge to comfort, console, or help the other person).These variables could be used to further disentangle different interpersonal processes and emotional or cognitive dispositions related to or intertwined with emotional contagion in the context of dyadic online video conferences. Besides, given that the present study focused on online video interaction, studies comparing the reported findings with in-person face-to-face interaction could provide additional insights as well as studies investigating online video interaction with the video cameras turned off (i.e., only transmitting the audio signal), based on recent findings that emphasized the relevance of video camera use for the emotional experiences during video conferences (Schwab et al., 2022).

## Conclusion

The present work adds to the existing research on emotional contagion in dyadic social interaction in several ways. First, we found clear patterns of emotional contagion for the emotions anger, joy, and sadness using both self-report and facial expression data. Second, we found robust empirical evidence for above-chance temporal co-occurrence of facially expressed joy between the two interaction partners. Third, our experimental setup and structured social interaction paradigm proved to be feasible and a promising foundation for future research on social interaction in online video conference settings as well as face-to-face social interaction. We conclude that emotions can be transmitted across interaction partners during online video interactions, but the face does not seem to be the key channel for those contagion processes, particularly not for negative emotions.

## Data Availability

The datasets presented in this study can be found in a publicly available online repository under https://osf.io/rabv7/ together with all reproducible data analysis scripts.

## Footnotes

^1^ www.meet.lrz.de

^2^ www.jitsi.org

^3^See www.doku.lrz.de for further technical information.

^4^www.imotions.com; Version 8.
